# Comparative analysis of the addition of empagliflozin versus doubling the furosemide dose in decompensated heart failure

**DOI:** 10.1007/s10557-024-07593-x

**Published:** 2024-06-12

**Authors:** Fuat Polat, Zeynettin Kaya, Cuma Süleymanoğlu

**Affiliations:** 1 Department of Cardiology, Health Sciences University Dr Siyami Ersek Thoracic and Cardiovascular Surgery Training and Research Hospital, Istanbul, Turkey; 2 Department of Cardiology, ASV Yaşam Hospital, Antalya, Turkey; 3 Department of Cardiology, Osmaniye State Hospital, Osmaniye, Turkey

**Keywords:** Decompensated heart failure, Empagliflozin, Furosemide, Echocardiographic outcomes, Rehospitalization

## Abstract

**Introduction:**

This study aims to compare the addition of SGLT2 inhibitors or doubling the diuretic dose in patients receiving treatment with beta-blockers, angiotensin-converting enzyme inhibitors (ACEi), or angiotensin receptor blockers (ARB), as well as mineralocorticoid receptor antagonists (MRA), for heart failure with reduced ejection fraction (HFrEF) who present to the emergency department with decompensated heart failure.

**Methods:**

This study is a single-center and prospective analysis. A total of 980 decompensated heart failure (HFrEF) patients receiving optimal medical therapy (OMT) according to the 2021 European heart failure guidelines were randomized in a 2:1 ratio into the furosemide and empagliflozin treatment arms. The analysis includes patient clinical characteristics, laboratory results, and echocardiographic data. Factors influencing rehospitalization were identified through multivariate Cox regression analysis. Log-rank analysis was employed to assess factors affecting rehospitalization.

**Results:**

The mean age of the patients was 67.9 years, with 52.1% being men. There was no significant impact of demographic, clinical, or echocardiographic factors on rehospitalization at 1 month; only the effect of treatment subgroups on rehospitalization was observed (*p* = 0.039). Significant echocardiographic and clinical improvements were seen in both treatment arms. The empagliflozin group exhibited significant improvements in 6-min walk distance, heart rate, body weight, NT-pro BNP levels, and eGFR level compared to the furosemide group. The rate of rehospitalization in the first month was significantly lower in those receiving empagliflozin (28.7%) compared to those receiving a double dose of furosemide (40.2%) (log-rank *p* = 0.013).

**Discussion and conclusion:**

This study provides valuable insights into the management of decompensated HFrEF and demonstrates that SGLT2 inhibitors offer benefits beyond glycemic control in this patient group. The significant reduction in rehospitalization rates and improvements in echocardiographic parameters underscore the potential of SGLT2 inhibitors in reducing acute heart failure episodes.

## Introduction

Heart failure represents not a disease in itself but rather a syndrome characterized by a combination of signs and symptoms stemming from the heart’s inability to effectively pump blood to support the circulatory system, whether at rest or during physical activity [1]. This failure manifests as impaired filling and elevated intracardiac pressure, leading to fluid retention in veins and tissues, resulting in edema due to fluid accumulation. Treatment primarily revolves around symptom improvement and halting disease progression. Attention must also be given to addressing reversible causes of heart failure. Therapeutic approaches encompass lifestyle modifications, pharmacological interventions, and, on occasion, various forms of device-based therapies. In rare instances, cardiac transplantation becomes necessary for end-stage heart failure. As of 2021, the standard of care for heart failure with reduced ejection fraction (HFrEF) involves quadruple medical therapy combining angiotensin receptor-neprilysin inhibitors (ARNI), beta-blockers (BB), mineralocorticoid receptor antagonists (MRA), and sodium/glucose cotransporter 2 inhibitors (SGLT2 inhibitors) [2].

Sodium/glucose cotransporter 2 inhibitors, also known as gliflozins or flozins, represent a class of medications that modulate sodium-glucose transport proteins within the nephron. Their primary metabolic effect involves inhibiting glucose reabsorption in the kidneys, thus reducing blood sugar levels [3]. SGLT2, the principal transport protein responsible for reabsorbing glucose from the glomerular filtrate back into circulation, accounts for approximately 90% of glucose reabsorption in the kidneys. SGLT2 is predominantly expressed in the kidneys’ epithelial cells lining the initial segment of the proximal convoluted tubule. By inhibiting SGLT2, gliflozins prevent the kidney’s reuptake of glucose from the glomerular filtrate, subsequently lowering blood glucose levels and promoting the excretion of glucose in the urine (glucosuria) [4].

Recent groundbreaking clinical trials have demonstrated that SGLT2 inhibitor therapies not only enhance blood glucose control but also reduce cardiovascular events and hospitalizations due to heart failure in patients with type 2 diabetes [5]. Remarkably, these clinical benefits have extended to patients with heart failure who do not have type 2 diabetes, although the precise underlying mechanisms remain unclear. Potential pathways implicated in these benefits include improved glycemic control, diuresis, weight reduction, and blood pressure reduction, though none individually account for the observed improvements in clinical outcomes. More recently, novel mechanisms have emerged to explain these benefits, encompassing enhanced cardiomyocyte calcium handling, improved myocardial energetics, induced autophagy, and reduced epicardial fat [6]. Notably, the ‘Dapagliflozin in Patients with Heart Failure and Reduced Ejection Fraction’ (DAPA-HF trial) and the ‘Empagliflozin Outcome Trial in Patients with Chronic Heart Failure with Reduced Ejection Fraction’ (EMPEROR-Reduced) trial have illustrated that the risk reduction observed in their primary endpoints is predominantly driven by a decrease in heart failure hospitalizations [7, 8]. A meta-analysis of these two trials indicates consistent effects of both dapagliflozin and empagliflozin, with reductions in all individual endpoints, including all-cause mortality, cardiovascular mortality, hospitalization for heart failure, or a decline in renal function. Thus, these remarkable benefits appear to represent a class effect of SGLT2 inhibitor therapies [9].

The primary outcome of the study was to compare rehospitalization for exacerbation of heart failure within the first month following emergency department admission across treatment subgroups. We applied this design to specifically evaluate the impact of these treatment strategies on short-term clinical stability in a real-world setting. This approach allowed us to directly evaluate the effectiveness of intensified diuretic therapy versus the addition of empagliflozin in the treatment of acute decompensated heart failure. Secondary outcomes were comparison of the effect of treatment groups on changes in echocardiographic parameters, 6-min walk distance (6 MWD), and heart rate. These results are intended to provide a holistic view of the patient’s clinical course and the potential benefits and risks associated with initial treatment decisions made during the acute phase of decompensated heart failure.

## Methods

This study is a single-center and prospective observational study conducted between October 2021 and December 2023 in patients who meet the diagnostic criteria in the 2021 heart failure guidelines of the European Society of Cardiology (ESC). Patients were included in the furosemide and empagliflozin group in a 2:1 ratio. The study recruited 980 patients with a confirmed diagnosis of HFrEF, according to specific inclusion criteria. Eligible participants were those diagnosed with HFrEF, defined by an ejection fraction of 40% or less, who were stabilized on guideline-directed medical therapy (GDMT) prior to enrollment. Patients were considered for inclusion if they presented to the emergency department (ER) with symptoms of acute decompensated heart failure related to volume overload, such as shortness of breath and edema, indicative of the need for immediate clinical intervention.

At the time of enrollment, all patients were on a regimen comprising ACE inhibitors (ACEi) or angiotensin receptor blockers (ARBs), BB, MRA, and furosemide, in line with the current guidelines. It is important to note that ARNI were not included in the standard treatment regimen for these patients. This omission was oeing to ARNI not being widely available for reimbursement in our country during the years of this study, limiting its accessibility and use in the target population.

Following an episode of decompensated heart failure, the initial treatment strategy within the first 24 h was to either double the furosemide dose or add furosemide 40 mg. After the first 24 h, approximately two-thirds of patients continued with the intensified furosemide therapy. The remaining one-third of the patients initiated empagliflozin in their treatment regimen, and furosemide therapy was adjusted back to the pre-decompensation dosage. This treatment approach aimed to manage fluid overload and improve cardiac function in these patients. However, to ensure the study’s focus on specific patient populations and to minimize confounding factors, certain criteria were established for patient inclusion and exclusion.

The differences observed between treatment groups may be influenced by the contrast between continuous diuretic treatment and the optimization of therapy by reducing diuretics and introducing SGLT2 inhibition [10]. Current heart failure guidelines recommend a nuanced approach to diuretic use, where in-hospital diuretic therapy is often necessary to manage acute fluid overload, but outpatient management aims to minimize diuretic use to avoid adverse effects and maintain stability [11, 12]. Guideline-directed medical therapy (GDMT) for heart failure emphasizes the use of diuretics primarily for symptom control and fluid management during acute decompensations, while SGLT2 inhibitors, such as empagliflozin, are increasingly recommended for their benefits on cardiac and renal outcomes, independent of their glycemic effects.

Patients with concurrent infection were excluded owing to the potential impact of active infections on fluid status and overall outcomes. Similarly, patients with acute coronary syndrome were excluded because the acute cardiac event could significantly influence the response to treatment and complicate the interpretation of results. Severe valvular disease was another exclusion criterion, likely chosen to maintain a more homogenous study population without the added complexity of valvular abnormalities affecting heart failure management.

Additionally, patients with stage 4 or higher renal failure were excluded because advanced renal impairment can significantly alter treatment strategies and outcomes in heart failure patients. Similarly, individuals diagnosed with heart failure with moderate (HFmrEF) or preserved (HFpEF) ejection fraction were excluded to focus specifically on patients with reduced ejection fraction heart failure. Patients whose heart failure treatment doses, other than diuretics and empagliflozin, were changed during the first 1-month follow-up of the patients were also not included in the study.

The study initially included a total of 1366 patients meeting the inclusion criteria. However, after applying the exclusion criteria, and propensity matching score the study continued with the remaining 980 patients who formed a more homogeneous cohort for analysis. The sample size for this study was determined using a power analysis based on the primary outcome of rehospitalization rates within the first month. The power analysis was performed using G*Power software, considering an alpha level of 0.05, power of 0.80, and an estimated effect size derived from previous literature. Specifically, the effect size was estimated on the basis of the anticipated difference in rehospitalization rates between the empagliflozin and furosemide treatment groups. The calculation yielded a required sample size of 980 patients, with a 2:1 ratio between the two treatment groups to ensure sufficient statistical power for subgroup analyses and to account for potential dropouts. This sample size determination method is commonly employed in clinical studies to ensure that the study has adequate power to detect meaningful differences or effects while minimizing the risk of type I and type II errors.

The process of matching involved several steps to ensure that the furosemide and empagliflozin subgroups were comparable. Age, gender, comorbid diseases such as diabetes or hypertension, and echocardiographic features were considered in the matching process. Propensity matching score techniques were used to equalize these factors between the two groups, aiming to reduce bias and improve the reliability of the study findings.

This comprehensive study also entailed a detailed analysis of various patient characteristics at baseline, including age, gender, vital signs at hospitalization, place of residence, existing comorbidities, medications upon hospital admission, laboratory results, and echocardiographic data. Any interruption in drug therapy, defined as a discontinuation or dose reduction for more than three days, was meticulously recorded.

Echocardiography, HR and 6 MWT evaluations were performed after 30 days of treatment in both groups. All echocardiographic measurements in our study were performed by one of two different cardiologists, both experienced in echocardiography, at a single center. To ensure unbiased results, the cardiologists conducted the imaging independently, without knowledge of the patients’ treatment arms. This blinding was crucial to minimize bias and enhance the validity of our findings. The measurements were conducted in a central echocardiography laboratory, ensuring consistency and accuracy in the data collection process. The echocardiographic evaluations focused on several parameters: left ventricular ejection fraction (LVEF) calculated using Simpson’s method, estimated right ventricular systolic pressure (RVSP) via the trans tricuspid pressure gradient, and diastolic function indicators such as the ratio of early transmitral flow velocity to mitral annular velocity (E/A). Additional parameters included tricuspid annular plane systolic excursion (TAPSE), heart rate (HR), and 6 MWD. These measurements were all conducted using advanced ultrasound systems from Siemens Medical Solutions.

### Statistical analysis

Statistical analysis was performed using appropriate tests for continuous and categorical variables. Baseline characteristics were compared between the furosemide and empagliflozin treatment groups using t tests for continuous variables and chi-square tests for categorical variables. The study’s analytical framework utilized multivariate Cox regression analysis to explore various factors influencing readmission within the first month post-discharge for all patients. Moreover, the impact of the initial treatment modifications on rehospitalization rates at the 1-month mark was evaluated for both patient groups through log-rank analysis. The groups were initially matched using propensity matching score analysis to ensure comparability in baseline characteristics. All statistical analyses were two-tailed, and a *p* value of less than 0.05 was considered statistically significant. Statistical analysis was performed using SPSS software version 25.0 (SPSS, Inc., Chicago, USA).

## Result

The study included 980 patients, of whom 52.1% were men, with an average age of 67.9 (years). Approximately half of the patients had hypertension (53.6%), and an equal number had diabetes (50%). Among those who presented with symptoms of volume overload, the furosemide treatment dose was doubled in 644 (65.7%) patients, while 336 (34.3%) patients received empagliflozin in addition to the treatment. Demographic, echocardiographic, and clinical characteristics of the patients, divided by treatment subgroups, are summarized in Table 1.

**Table 1:** Characteristics of the patients, including demographics, echocardiographic data, and clinical features, categorized on the basis of treatment subgroups

Patient characteristics	All patients (*N* = 980)	Patients with doubled furosemide dose (*N* = 644)	Patients with added empagliflozin treatment (*N* = 336)	*P* value
Age (years)	67.9 ± 11.1	68.9 ± 11.4	66.0 ± 10.5	0.14
Gender (male)	511 (52.1)	329 (51.1)	182 (54.2)	0.43
Body weight (kg)	80.41 ± 7.8	79.6 ± 6.9	81.3 ± 8.7	0.25
BMI (kg/m^2^)	27.5 ± 2.4	27.3 ± 2.7	27.8 ± 2.3	0.79
Hypertension	525 (53.6)	322 (50.0)	203 (60.4)	0.16
DM	490 (50.0)	308 (47.8)	182 (54.2)	0.30
Ischemic heart failure	726 (74.1)	472 (73.3)	254 (75.6)	0.36
Na (mEq/L)	133.8 ± 1.8	133.7 ± 1.9	134.1 ± 1.7	0.82
K (mmol/L)	4.4 ± 1.0	4.4 ± 0.8	4.4 ± 1.1	0.91
NT-pro BNP (pg/mL)	14,027 ± 1186	13,837 ± 1243	14,358 ± 1125	0.72
eGFR (mL/min/1.73 m^2^)	62.8 ± 24.1	63.3 ± 25.7	62.1 ± 21.4	0.56
EF %	29.7 ± 5.4	30.0 ± 5.3	29.0 ± 5.6	0.28
Mitral inflow E/A	0.8 ± 0.1	0.8 ± 0.1	0.8 ± 0.2	0.64
Mitral tissue Doppler E/e’	9.3 ± 1.4	9.4 ± 1.4	9.3 ± 1.5	0.86
TAPSE (mm)	13 ± 2.0	13.2 ± 2.0	12.7 ± 2.0	0.16
TRV (m/sn)	3.0 ± 0.5	3.0 ± 0.5	2.9 ± 0.5	0.35
6 MWD (m)	284 ± 31	285 ± 33	283 ± 27	0.78
Heart rate (beats per min)	86 ± 5	87 ± 5	85 ± 6	0.10
Final furosemide dose (mg)	48.2 ± 5.2	60.6 ± 8.1	28.8 ± 4.2	< 0.001

*BMI* body mass index, *DM* diabetes mellitus, *EF* ejection fraction, *GFR* glomerular filtration rate, *K* potassium, *6 MWD* 6 minute walk test, *Na* sodium, *NT pro-BNP* N-terminal pro B type natriuretic peptide, *TAPSE* tricuspid annular plane systolic excursion, *TRV* tricuspid regurgitation velocity

Factors contributing to rehospitalization within the first month were assessed through multivariate Cox regression analysis. Remarkably, no significant impact of demographic, clinical, or echocardiographic factors on rehospitalization was observed, except for the treatment group (*p* = 0.039), as shown in Table 2.

**Table 2:** Multivariate Cox regression analysis demonstrating the impact of patient characteristics on rehospitalization in patients with decompensated heart failure

Patients characteristics	Hazard ratio	95% confidence interval	*P* value
Age	1.00	0.98–1.03	0.73
Gender	0.95	0.51–1.77	0.87
BMI	1.17	0.63–2.12	0.65
Hypertension	1.12	0.57–2.21	0.73
DM	1.26	0.60–2.64	0.54
EF%	1.04	0.91–1.19	0.54
Mitral inflow E/A	0.14	0.01–4.30	0.26
Mitral tissue Doppler E/e’	1.23	0.81–1.89	0.34
TAPSE	1.06	0.82–1.36	0.67
TRV	0.92	0.84–1.02	0.10
6 MWD	1.00	0.99–1.01	0.94
HR	1.03	0.96–1.10	0.37
Treatment group*	2.2	1.04–4.74	0.039*

*BMI* body mass index, *DM* diabetes mellitus, *6 MWD* 6 minute walk test, *TAPSE* tricuspid annular plane systolic excursion, *TRV* tricuspid regurgitation velocity

^*^furosemide vs. empagliflozin

**Fig. 1:**
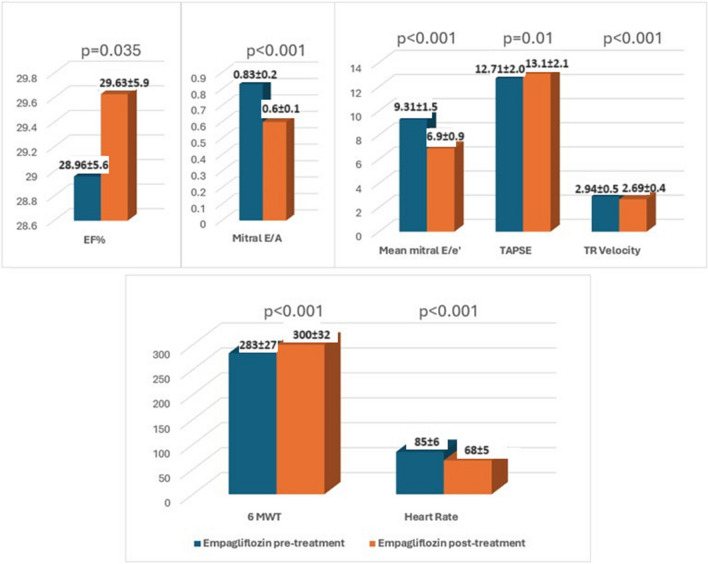
Alterations in echocardiographic parameters, 6-min walk test, and heart rate observed before and after the administration of empagliflozin treatment

**Fig. 2:**
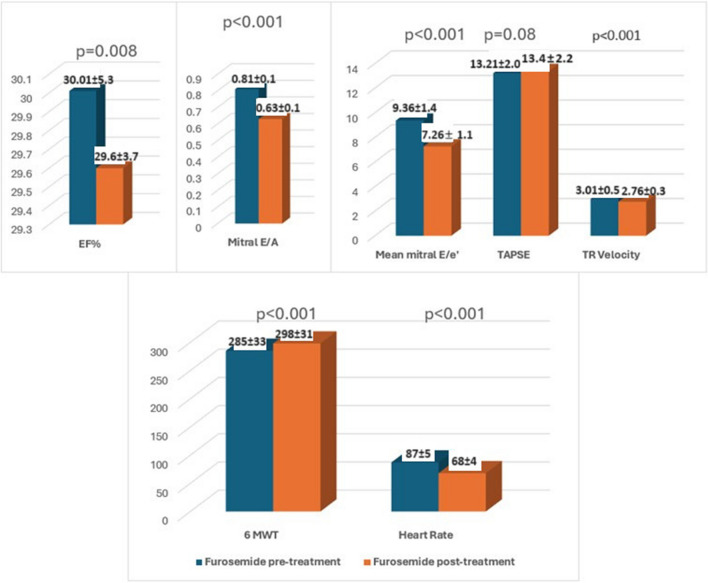
Alterations in echocardiographic parameters, 6-min walk test, and heart rate observed before and after the administration of furosemide treatment

**Fig. 3:**
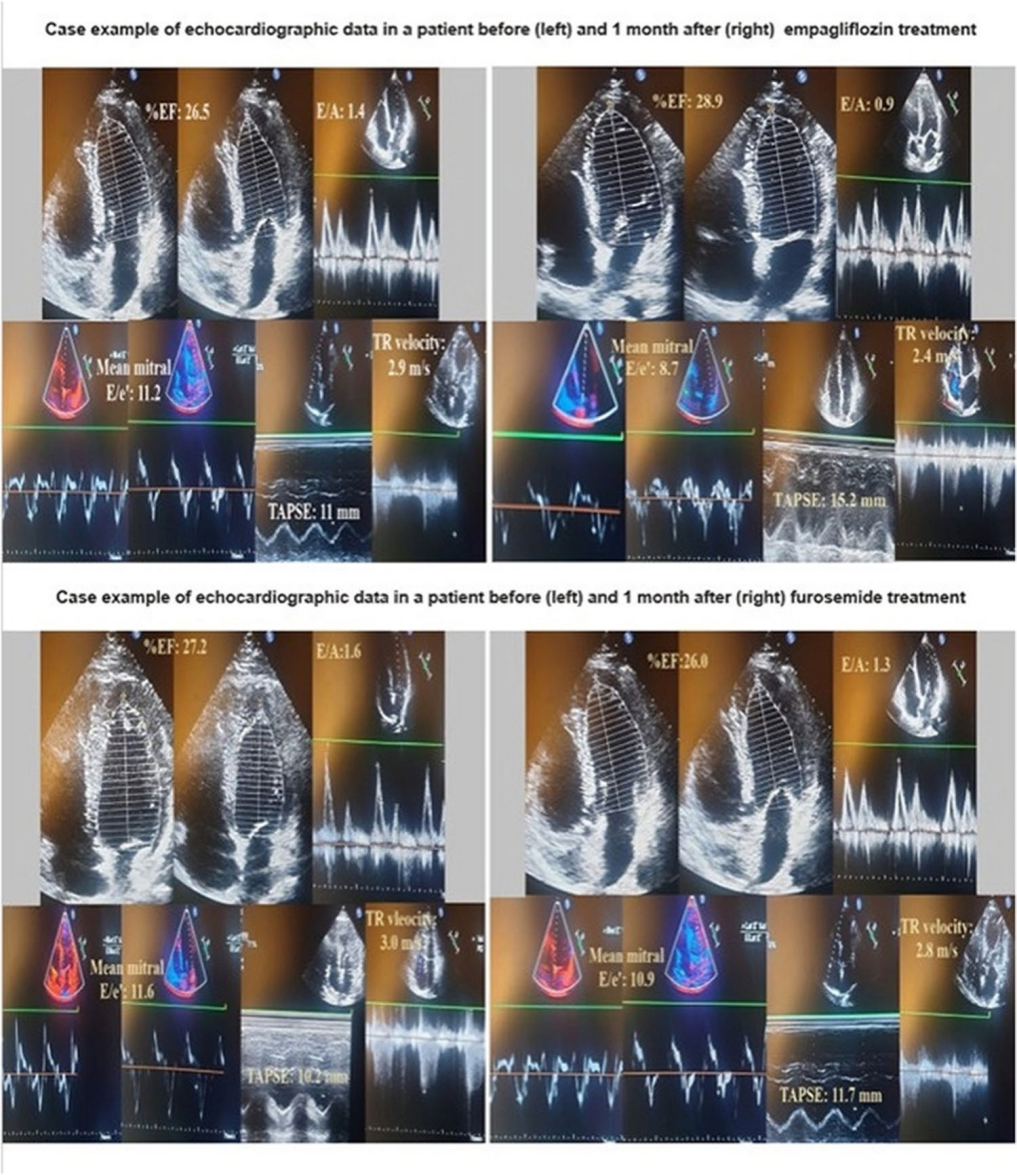
An example of echocardiographic data in one patient at pretreatment and 1 month post-treatment in the empagliflozin and furosemide treatment groups

Echocardiographic and clinical parameters were assessed in both the empagliflozin and furosemide treatment groups at the end of the first month. There was no severe mitral annular calcification in the patients included in the study. In the empagliflozin group, significant reductions were observed in the mitral E/A ratio, mitral Doppler tissue E/e’ ratio, and tricuspid regurgitation velocity (TRV). Furthermore, a significant increase in 6 MWT and a decrease in HR were documented at a fixed dose of beta-blockers (Fig. 1). Similarly, significant reductions in mitral E/A ratio, mitral Doppler tissue E/e’ ratio, and TRV were observed in the group with escalated furosemide dosage. In addition, a significant increase in 6 MVT and a decrease in HR were observed (Fig. 2). An example of echocardiographic data in one patient at pretreatment and 1 month post-treatment in the empagliflozin and furosemide treatment groups is shown in Fig. 3.

Body weight, pro-BNP, Na, K, and eGFR values were similar in both groups at baseline. The values of these parameters before treatment and 1 month after treatment were compared in both groups. In patients with doubled furosemide dose, a significant change was observed in all parameters examined at the end of the 1-month follow-up. While no significant change was observed in electrolyte values in the patients with added empagliflozin treatment group, a significant change was observed in body weight, pro-BNP, and eGFR levels (Table 3).

**Table 3:** Pre-treatment and post-treatment change in patient characteristics except echocardiographic parameters, 6 MWT, and HR

Patient characteristics	Patients with doubled furosemide dose		*P* value	Patients with added empagliflozin treatment		*P* value
	Pre-treatment	Post-treatment		Pre-treatment	Post-treatment	
Body weight (kg)	79.6 ± 6.9	76.0 ± 5.6	0.003	81.3 ± 8.7	75.7 ± 7.9	< 0.001
Na (mEq/L)	133.7 ± 1.9	131.9 ± 2.1	0.045	134.1 ± 1.7	133.3 ± 1.6	0.420
K (mmol/L)	4.4 ± 0.8	4.0 ± 0.7	0.002	4.4 ± 1.1	4.3 ± 0.8	0.088
NT-pro BNP (pg/mL)	13,837 ± 1243	984 ± 341	< 0.001	14,358 ± 1125	984 ± 341	< 0.001
eGFR (mL/min/1.73 m^2^)	63.3 ± 25.7	68.7 ± 24.3	< 0.001	62.1 ± 21.4	69.2 ± 21.9	< 0.001

*GFR* glomerular filtration rate, *HR* heart rate, *K* potassium, *6 MWD* 6 min walking taste, *Na* sodium, *NT pro-BNP* N terminal pro B type natriuretic peptide

The comparison of echocardiographic and clinical characteristics at 1 month revealed significant improvements in the empagliflozin group compared to the furosemide group. Specifically, the empagliflozin group showed significant increases in EF% and mitral inflow E/A, along with improved TAPSE (*p* < 0.001, *p* = 0.028, and *p* = 0.011, respectively). Additionally, the empagliflozin group exhibited a more pronounced reduction in body weight (*p* = 0.002), less change in Na value (*p* = 0.045), and a greater decrease in NT-pro BNP (pg/mL) levels (*p* = 0.02). Moreover, there was a more significant improvement in eGFR levels in the empagliflozin group (*p* < 0.001) (Table 4).

**Table 4:** The comparison of treatment groups for changes in echocardiographic and clinical characteristics at 1 month

Patient characteristics	Patients with doubled furosemide dose		Patients with added empagliflozin treatment		*P* value
	Pre-treatment	Delta change	Pre-treatment	Delta change	
EF %	30.01 ± 5.3	−0.41	28.96 ± 5.6	0.67	< 0.001
Mitral inflow E/A	0.81 ± 0.1	−0.18	0.83 ± 0.2	−0.23	0.028
Mitral tissue Doppler E/e’	9.36 ± 1.4	−2.1	9.31 ± 1.5	−2.41	0.49
TAPSE (mm)	13.21 ± 2.0	0.19	12.71 ± 2.0	0.39	0.011
TRV (m/sn)	3.01 ± 0.5	−0.25	2.94 ± 0.5	−0.25	0.96
6 MWD (m)	285 ± 33	13	283 ± 27	17	0.30
Heart rate (beats per minute)	87 ± 5	−19	85 ± 6	−17	0.52
Body weight (kg)	79.6 ± 6.9	−3.6	81.3 ± 8.7	−5.6	0.002
Na (mEq/L)	133.7 ± 1.9	−1.8	134.1 ± 1.7	−0.8	0.045
K (mmol/L)	4.4 ± 0.8	−0.4	4.4 ± 1.1	−0.1	0.35
NT-pro BNP (pg/mL)	13,837 ± 1243	−12,853	14,358 ± 1125	−13,374	0.02
eGFR (mL/min/1.73 m^2^)	63.3 ± 25.7	5.4	62.1 ± 21.4	7.1	< 0.001

*GFR* glomerular filtration rate, *HR* heart rate, *K* potassium, *6 MWD* 6 min walking taste, *Na* sodium, *NT pro-BNP* N terminal pro B type natriuretic peptide, *TAPSE* tricuspid annular plane systolic excursion, *TRV* tricuspid regurgitation velocity

**Fig. 4:**
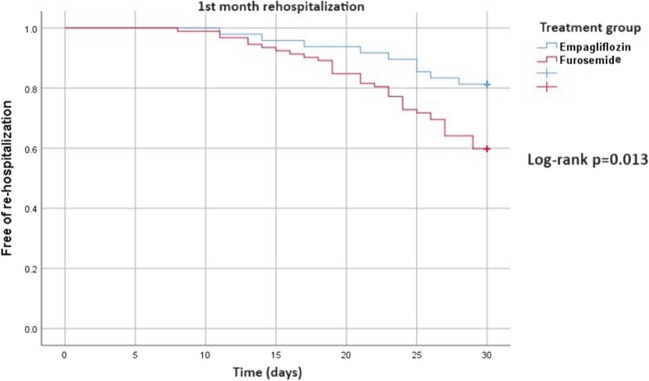
The comparison of rehospitalization between the groups receiving empagliflozin and those receiving a doubled dose of furosemide using log-rank survival analysis

Rehospitalization was observed after 1 month of follow-up in 63 patients (28.7%) treated with empagliflozin and 259 patients (40.2%) treated with a doubled dose of furosemide. The most common reason for rehospitalization in both groups was worsening heart failure (44 patients [70%] in the empagliflozin group and 194 patients [74.9%] in the furosemide group, respectively). The rehospitalization rate in the first month was statistically significantly lower in patients receiving empagliflozin treatment (log-rank *p* = 0.013) (Fig. 4). At 1-month follow-up, deaths were reported in seven patients, four in the empagliflozin group and three in the furosemide group. There was no statistical significance between groups in terms of 1-month mortality.

## Discussions

Heart failure, a complex clinical syndrome, poses a significant burden on healthcare systems worldwide [13, 14]. The current study aimed to compare the effects of adding empagliflozin or doubling the dose of loop diuretics in patients with HFrEF who presented to the emergency department with decompensated heart failure. The findings shed light on the potential benefits of SGLT2 inhibitors beyond their established role in glycemic control, extending to improved echocardiographic and clinical outcomes compared to traditional diuretic escalation.

The study’s cohort comprised individuals diagnosed with HFrEF already receiving GDMT, including ACEi or ARBs, beta-blockers, and MRA. Furosemide, a loop diuretic, was the standard diuretic therapy [15]. During the study, patients experiencing decompensated heart failure were randomized into two groups: one receiving empagliflozin, an SGLT2 inhibitor, in addition to GDMT, and the other with a doubled dose of furosemide.

Echocardiographic parameters, including LVEF, estimated RVSP), and the ratio of mitral (E/A), were evaluated as key indicators of cardiac function [16]. Additionally, clinical outcomes such as the 6 MWD distance and HR were assessed. The results demonstrated that both treatment arms exhibited significant improvements in various echocardiographic parameters and clinical outcomes at the end of the 1-month follow-up [17, 18].

Interestingly, the empagliflozin group displayed notable reductions in the mitral E/A ratio, mitral Doppler tissue E/e’ ratio, and TRV, suggesting improved left ventricular filling pressure and reduced right ventricular pressure. Furthermore, the increase in 6 MWD distance and decrease in HR in this group indicated enhanced exercise tolerance and reduced sympathetic activity. This conclusion was not confirmed by direct measurement of sympathetic activity parameters. While sympathetic activity can be assessed by various methods, such as measuring plasma levels of catecholamines such as norepinephrine or using heart rate variability analysis, the observed changes in 6 MWD and HR may be indirect indicators of improved exercise capacity and reduced sympathetic tone without direct measurement of sympathetic activity. Similar improvements were observed in the group where the furosemide dose was increased, highlighting the effectiveness of both interventions in alleviating cardiac strain and enhancing functional capacity.

Importantly, the study’s primary endpoint focused on rehospitalization within the first month, a crucial metric in evaluating the efficacy of interventions in managing acute heart failure exacerbations [19]. The results demonstrated a statistically significant reduction in rehospitalization rates for patients receiving empagliflozin compared to those with a doubled diuretic dose. Our study’s results are consistent with other important studies, including the ’Dapagliflozin in Patients with Heart Failure and Reduced Ejection Fraction’ (DAPA-HF) trial, the ‘Empagliflozin Outcome Trial in Patients with Chronic Heart Failure with Reduced Ejection Fraction’ (EMPEROR-Reduced) trial, and the EMPULSE study comparing empagliflozin treatment with placebo after stabilization in acute heart failure patients. These trials collectively emphasize the efficacy of SGLT2 inhibitors in reducing heart failure-related hospitalizations and improving clinical outcomes [7, 8, 20–22]. Additionally, studies investigating dapagliflozin treatment during acute decompensated heart failure have shown significant weight loss and lower diuretic doses during hospitalization, while studies involving early addition of empagliflozin to standard diuretic therapy have demonstrated increased urine output without impacting renal function in acute heart failure patients [23, 24]. Moreover, research has indicated that empagliflozin treatment reduces markers of tubular injury in patients with acute decompensated heart failure, further supporting the beneficial effects of SGLT2 inhibitors in this patient population [25].

The reduced hospitalization rate observed in the SGLT2 inhibitor (SGLT2-I) group can be attributed primarily to the effect of empagliflozin rather than the doubling of the furosemide dose. This conclusion is supported by several key findings in the study. First, the empagliflozin group showed significant improvements in echocardiographic parameters such as the mitral E/A ratio, mitral Doppler tissue E/e’ ratio, and tricuspid regurgitation velocity (TRV), indicating enhanced cardiac function and reduced ventricular pressures. Additionally, the empagliflozin group demonstrated notable reductions in rehospitalization rates compared to the group with escalated furosemide dosage, aligning with previous trials highlighting the efficacy of SGLT2 inhibitors in reducing heart failure-related hospitalizations. These findings collectively suggest that empagliflozin plays a substantial role in mitigating acute heart failure episodes and contributing to the observed decrease in rehospitalization rates.

High-dose loop diuretics may potentially have a negative impact on outcomes in patients with heart failure [26, 27]. While loop diuretics are essential for managing volume overload and symptoms of heart failure exacerbations, their high doses can lead to electrolyte imbalances, renal dysfunction, and neurohormonal activation, contributing to adverse events such as worsening renal function, electrolyte disturbances, and neurohormonal activation, which are associated with increased morbidity and mortality in heart failure patients. Therefore, careful dose titration and monitoring of electrolytes and renal function are crucial to optimizing the therapeutic benefits of loop diuretics while minimizing their potential adverse effects on outcomes.

In heart failure management, the decision regarding the dosage of diuretics depends on the clinical context, including the severity of symptoms, volume status, renal function, and response to initial treatment. In acute decompensated heart failure cases requiring recompensation, an increased dosage of diuretics may be necessary to achieve adequate volume reduction and symptom relief. This approach aims to address acute volume overload and congestion promptly. On the other hand, in stable outpatient settings, a lower dosage of diuretics is often preferred to maintain euvolemia while minimizing the risk of electrolyte imbalances, renal impairment, and neurohormonal activation associated with high-dose diuretic therapy. Tailoring diuretic dosing strategies to individual patient needs and closely monitoring response and tolerability are essential aspects of optimizing heart failure management [28].

The study’s reliance on data from a single center may limit the generalizability of the results to broader patient populations across different healthcare settings. Future research should aim for multicenter studies to validate the findings in diverse patient cohorts. The relatively short 1-month follow-up period might not capture long-term outcomes and sustainability of treatment effects. Extending the follow-up duration in future studies could provide insights into the durability of improvements observed. The study primarily focused on empagliflozin, and while it provided valuable insights into this specific SGLT2 inhibitor, caution should be exercised when extrapolating these findings to other SGLT2 inhibitors. Future studies comparing different SGLT2 inhibitors could elucidate potential differences in efficacy and safety profiles.

The study’s findings highlighted the extended benefits of SGLT2 inhibitors beyond glycemic control, showing improvements in echocardiographic parameters, clinical outcomes, and reduced rehospitalization rates compared to traditional diuretic escalation. This expands the understanding of SGLT2 inhibitors in managing heart failure patients with reduced ejection fraction. The observed reductions in mitral E/A ratio, mitral Doppler tissue E/e’ ratio, and TRV in the empagliflozin group provide mechanistic insights into improved cardiac function, reduced ventricular pressures, enhanced exercise tolerance, and reduced sympathetic activity. These mechanistic findings contribute to a deeper understanding of the effects of SGLT2 inhibitors on heart failure pathophysiology.

Conducting multicenter studies involving diverse patient populations and healthcare settings can enhance the external validity and generalizability of findings. Extending the follow-up duration to assess long-term outcomes, including mortality and major adverse cardiovascular events, can provide a comprehensive understanding of the sustained effects of interventions. Considering comparative studies among different SGLT2 inhibitors and other heart failure therapies can elucidate potential differences in efficacy, safety, and tolerability profiles, guiding clinical decision-making.

By acknowledging these limitations, emphasizing the novelty of findings, and providing advice for future research directions, the study’s contributions to the field of heart failure management can be effectively contextualized and further advanced.

## Conclusion

In conclusion, this study adds significant insights into the management of heart failure with reduced ejection fraction (HFrEF) by comparing the effects of empagliflozin addition versus doubling the dose of loop diuretics in patients presenting with acute decompensated heart failure. The findings underscore the multifaceted benefits of SGLT2 inhibitors beyond glycemic control, showcasing improvements in echocardiographic parameters, clinical outcomes, and notably, reduced rehospitalization rates compared to traditional diuretic escalation. Mechanistic insights into improved cardiac function, reduced ventricular pressures, enhanced exercise tolerance, and reduced sympathetic activity were observed in the empagliflozin group, contributing to a deeper understanding of SGLT2 inhibitors’ effects on heart failure pathophysiology. These findings support the growing body of evidence advocating for the incorporation of SGLT2 inhibitors into the standard of care for HFrEF, emphasizing the need for further multicenter studies, longer follow-up periods, and comparative analyses among different SGLT2 inhibitors to refine treatment strategies and optimize patient outcomes in heart failure management.

## Data Availability

All relevant data supporting the findings of this study are available upon request and will be provided by the corresponding author.

## Code Availability

The research presented in this manuscript does not involve the development or utilization of any custom software applications or specific code. As such, there is no code availability associated with this study. The analysis and findings rely on standard statistical methods and commercially available software tools. For any inquiries related to the methodology or data analysis, please contact the corresponding author.
